# Supplementary material for: Complexome profiling of the Chlamydomonas psb28 mutant reveals TEF5 as an early PSII assembly factor
Source: Plant Cell. 2025 Mar 17;37(6):koaf055. doi: 10.1093/plcell/koaf055 (PMC12164590; doi:10.1093/plcell/koaf055)
Supplement: koaf055_Supplementary_Data [file koaf055_supplementary_data.zip › psb28_interactive.zip!/plots/intensity/qualityPlots/peptides/ATPVE_Cre17.g734500.t1.2.html]

### Cre17.g734500.t1.2

Trivial name: ATPVE  
SeqCoverage [median]: 48.30  
Mass: 26.24 kDa  
  
 

| Sequence | MissedCleavage | Mass [Da] | wtIntensity | muIntensity | Score |
| --- | --- | --- | --- | --- | --- |
| AKLFGVVAQGQH | 1 | 1253.69 | 1408.20 | 9443.44 | 82.17 |
| EAAVQDIITDAK | 0 | 1272.66 | 18205.01 | 51970.78 | 196.48 |
| EAAVQDIITDAKAR | 1 | 1499.79 | 0.00 | 1583.14 | 51.73 |
| EGQVEVK | 0 | 787.41 | 525.25 | 4421.56 | 112.37 |
| EVVEPAR | 0 | 798.42 | 0.00 | 2748.60 | 105.39 |
| EVVEPARK | 1 | 926.52 | 364.98 | 1048.93 | 107.67 |
| FIKQEAEEK | 1 | 1120.58 | 5892.56 | 12367.94 | 174.41 |
| IAYQANLPAIR | 0 | 1228.69 | 1058.44 | 45314.76 | 186.30 |
| LFGVVAQGQH | 0 | 1054.56 | 5897.30 | 28773.72 | 129.40 |
| LLQDLLVQAMR | 0 | 1298.74 | 0.00 | 6831.06 | 128.60 |
| LLQDLLVQAMRK | 1 | 1426.83 | 0.00 | 2013.90 | 94.30 |
| LQLLEQEK | 0 | 999.56 | 2454.58 | 4688.87 | 122.19 |
| LQLLEQEKSK | 1 | 1214.69 | 0.00 | 1203.83 | 91.66 |
| MNEVEVER | 0 | 1004.46 | 6799.24 | 17306.54 | 115.49 |
| QVDLLLVK | 0 | 926.58 | 4210.84 | 10450.62 | 79.12 |
| VRQVDLLLVK | 1 | 1181.75 | 0.00 | 9332.11 | 93.10 |
| VSAEEEFNLEK | 0 | 1293.61 | 2229.09 | 6139.87 | 166.97 |
|  |  |  | 49045.5 | 215639.7 |  |
